# Structural and Textural Characteristics of 3D-Printed Protein- and Dietary Fibre-Rich Snacks Made of Milk Powder and Wholegrain Rye Flour

**DOI:** 10.3390/foods9111527

**Published:** 2020-10-23

**Authors:** Martina Lille, Anni Kortekangas, Raija-Liisa Heiniö, Nesli Sozer

**Affiliations:** VTT Technical Research Centre of Finland Ltd., P.O. Box 1000, FI-02044 VTT Espoo, Finland; anni.kortekangas@vtt.fi (A.K.); raija-liisa.heinio@outlook.com (R.-L.H.); nesli.sozer@vtt.fi (N.S.)

**Keywords:** food 3D printing, milk powder, wholegrain rye flour, baking, rheology, X-ray tomography, texture, sensory analysis

## Abstract

This study addressed the potential of 3D printing as a processing technology for delivering personalized healthy eating solutions to consumers. Extrusion-based 3D printing was studied as a tool to produce protein- and dietary fibre-rich snack products from whole milk powder and wholegrain rye flour. Aqueous pastes were prepared from the raw materials at various ratios, grid-like samples printed from the pastes at ambient temperature and the printed samples post-processed by oven baking at 150 °C. Printing pastes were characterized by rheological measurements and the baked samples by X-ray micro tomography, texture measurements and sensory analysis. All formulations showed good printability and shape stability after printing. During baking, the milk powder-based samples expanded to a level that caused a total collapse of the printed multiple-layer samples. Shape retention during baking was greatly improved by adding rye flour to the milk formulation. Sensory evaluation revealed that the volume, glossiness, sweetness and saltiness of the baked samples increased with an increasing level of milk powder in the printing paste. A mixture of milk powder and rye flour shows great potential as a formulation for healthy snack products produced by extrusion-based 3D printing.

## 1. Introduction

Extrusion-based 3D food printing is an emerging food manufacturing technology that enables food production tailored for individual needs and preferences. Early application of 3D food printing focused more on the aesthetics and shape, with various readily available materials (e.g., chocolate, hazelnut cream, cream cheese) as raw material [1,2,3]. However, recent research utilizes 3D printing as a potential processing technology to deliver personalized healthy eating solutions by the use of a wide range of protein and/or dietary fibre-rich materials, such as oat and faba bean protein concentrates [4], meat [5], milk protein [6] and soy protein isolate [7], either as such or in combination with starch or other hydrocolloids. On the other hand, 3D printing could potentially be used for the delivery of micronutrients, antioxidants and probiotics [8,9,10,11,12]. Moreover, a wide range of studies illuminate the possibilities of mimicking traditional foods, such as bread, cake or cookies, by 3D printing [13,14].

The formulation of printable “inks” from food raw materials can be a challenging task. The printing formulations have to fulfil at least two important requirements: they have to be extrudable through a nozzle within the extrusion force range defined by the printing device at hand and they have to “solidify” quickly enough after deposition to maintain the shape of the created object [15]. These kinds of properties can be generated by temperature-induced phase transitions in materials, such as chocolate [16], which is extruded at low viscosity in molten state and cooled quickly after deposition to solidify the material. Hydrocolloids that form low-viscous solutions at elevated temperatures and gel upon cooling can be utilized in 3D printing [17] in a similar way to chocolate, but precise temperature control is required in both cases. Printing can also be carried out at ambient temperature with formulations showing shear rate-dependent flow behaviour, i.e., with shear-thinning materials having a high viscosity at low shear forces and a gradually decreasing viscosity at shear rates above a critical value. There are many examples in the literature on successfully utilizing shear-thinning materials, such as hydrocolloid gels [18,19], tomato paste [20], mashed potatoes [21] and dough [22] in extrusion-based 3D printing. It has been suggested that rheological measurements could be utilized in predicting the suitability of food formulations for extrusion-based 3D printing [19,20,23].

From an engineering point of view, the study of printing parameters as well as printing methods gives important insights into the ultimate opportunities and constraints of food 3D printing. The optimization of printing parameters such as nozzle size or speed enables control of both the printing precision [13,24] and speed, which is a limiting factor in extrusion-based 3D food printing as reviewed by Sun et al. [25].

Another huge challenge in developing suitable food formulations for 3D printing is to ensure dimensional stability of the printed structures during and after post-processing by baking, which is often required for making cereal-based 3D-printed structures palatable. However, post-processing by baking at elevated temperatures might lead to shape instability or substantial deformation of printing patterns due to changes on micro- and macromolecular levels. Modifications in the printing paste composition, properties of individual ingredients or the post-processing process itself are often required to improve the dimensional stability of post-processed printed structures [26]. For example, according to Yang et al. [24], fast freezing of printed samples at −65 °C prior to baking at 190 °C allowed higher shape stability of low-gluten flour-based cookie dough. In another study, the inclusion of xanthan gum to wheat flour–based cookie dough improved the dimensional stability during baking at 170 °C, most likely by increasing the mechanical strength of the dough during the heating process [27].

The present study builds upon our previous work [4] aiming at developing formulations for extrusion-based 3D printing of healthy customized snack products. In the previous study, we showed that an aqueous paste of fat containing milk powder performed very well in 3D printing at ambient temperature, in terms of both printability and shape stability after printing and drying at 100 °C. The samples in that study, however, were printed at a low speed (2 mm/s) and fine resolution (0.41 mm nozzle) which made the production of printed structures very slow. The printed samples were also very small due to the low volume (3 mL) of the syringes applied in the advanced 3D-printing device. In the present work, we scaled up the printing process at higher printing speeds by using a commercial device specifically designed for food 3D printing. Wholegrain rye flour was added to the formulation at various ratios as a dietary fibre source. Samples were printed in a grid-like pattern in two heights (single and 5 layers), post-processed by oven baking at 150 °C, and further analysed for microstructural, textural and sensory properties. We showed that a mixture of milk powder with wholegrain rye flour showed benefits in terms of post-processing as compared with pure milk powder or rye flour formulations.

## 2. Materials and Methods

### 2.1. Materials

Commercial whole milk powder (Valio, Finland) consisting of 27% protein, 41% carbohydrates, 25% fat and finely milled wholegrain rye flour (RavintoRaisio Oy, Finland) consisting of 10% protein, 57% carbohydrates, 2.3% fat and 20% dietary fibre were used as raw materials for printing pastes.

### 2.2. Preparation of Pastes for 3D Printing

Pastes for 3D printing were prepared from rye flour and/or milk powder at ratios (Table 1) that were in preliminary experiments found to perform well in printing. Rye flour (R) and/or milk powder (M) pastes were formed by first dispersing the dry ingredients in water with an electric hand mixer (OBH Nordica, Denmark) equipped with one dough hook (3 × 20 s cycles at speed 1) and mixing was continued with a Bamix wand mixer (Switzerland) for three 10 s cycles at speed 2. Pastes were used for rheological characterization or printing right after preparation.

### 2.3. Rheological Characterization of Pastes

Viscoelastic properties of printing pastes were measured by stress sweep measurements with a Discovery HR-2 rheometer (TA Instruments, New Castle, DE, USA). The measurements were carried out at 22 °C at several time points up to 4 h after paste preparation. Stainless steel parallel plates with a diameter of 20 mm and a gap of 1.5 mm were used as measuring geometry. After sample loading and trimming, the edge of the sample was covered with rapeseed oil to prevent water evaporation and the sample was allowed to relax for 5 min before starting the measurement. The stress sweep measurements were carried out with a logarithmically increasing shear stress at a frequency of 0.1 Hz (start stress 0.01 Pa). Each mixture was measured in triplicate. The average storage modulus G’, loss modulus G’’ and phase angle from the linear viscoelastic region as well as yield stress (defined as the stress at which G’ falls below 90% of the G’ in the linear region) were extracted from the measurements.

### 2.4. 3D Printing and Post-Processing

Printing was performed at ambient temperature with a Foodini 3D printer (Natural Machines Inc., Seattle, WA, USA) using a printing capsule with a 1.5 mm diameter nozzle. The printed pattern was a grid consisting of 5 × 3 squares and external dimensions of approximately 67 × 40 mm. The printing path was automatically created by the printer as two-ply, i.e., the final shape consisted of two printed threads laid side-by-side. Both single-layer and 5-layer samples were printed. Single-layer samples were printed with paste mixtures R, R1M1 and M, whereas 5-layer samples were printed with all mixtures. The printing speed was 2100–2200 mm/min and the distance between layers 1.2 mm. Printing of 1-layer samples gave approximately 7 replicates from one batch of printing paste whereas printing of 5-layer samples gave 3 replicates from one batch of printing paste. Each batch of printing paste was used for printing within 4 h after paste preparation.

Immediately after printing, samples were photographed, weighed and post-processed by baking in a forced convection lab oven (OF-12, Jeio Tech, Daejeon, Korea) at 150 °C. The baking time was in preliminary trials determined to a level resulting in a final dry matter content of 92–94% after baking and storage. The single-layer samples were baked for 5 min and the 5-layer samples for 20 min (R and R3M1), 15 min (R1M1), 12 min (R1M3) or 10 min (M). Single-layer samples were, after baking, stored sealed in plastic bags in a cool room (16 °C) for 7–10 days prior to analysis (to enable sensory analysis). The 5-layer samples were, after baking, stored open in a room with controlled relative humidity (50% RH) and temperature (23 °C) for 7 days in order to equilibrate the moisture content of the samples prior to analysis.

### 2.5. Texture Analysis

The texture of the printed and baked samples was assessed by a cutting test as described by Lille et al. [4]. The samples were cut in halves (cutting position shown in Figure 3) with a knife blade attached to a Texture Analyser (Stable Micro Systems Ltd., Godalming, UK). The test speed was 2 mm/s. The maximum force at the breaking point of the sample was recorded as fracture force. The number of replicates was at least 17 for single-layer samples (prepared from 3 batches of printing paste) and 6 for 5-layer samples (prepared from 2 batches of printing paste).

### 2.6. Dry Matter Content

All samples were analysed for dry matter content by using a halogen moisture analyser (MB120, Ohaus, Greifensee, Switzerland) right after texture measurement.

### 2.7. Stereomicroscopy

The cross-sectional structure of baked samples was observed by a SteREO Discovery.V8 stereomicroscope equipped with an Achromat S 0.5 objective (Carl Zeiss MicroImaging GmbH, Göttingen, Germany) and imaged using a DP-25 single chip colour CCD camera (Olympus Life Science Europa GmbH, Hamburg, Germany).

### 2.8. Microstructural Analysis by X-ray Micro Tomography

**Figure 1:**
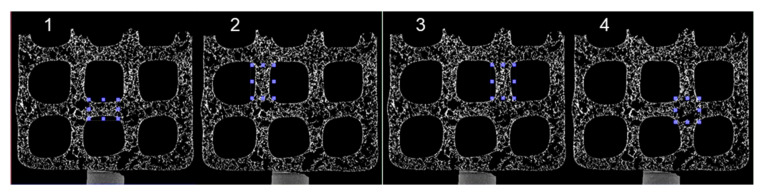
Parts of samples chosen for 3D pore morphology analysis by X-ray micro tomography (marked with blue in images 1–4).

The micro-structures of single layer printed samples were analysed by X-ray micro tomography (XMT) using a GE phoenix v|tome|x s 240 desktop XMT system (GE Sensing and Inspection Technologies GmbH, Wunstorf, Germany). The X-ray tube was operated at a voltage of 50 kV and a current of 440 µA and XMT data was collected using a 12-bit CCD camera (2024 × 2024 pixels). Half-cut samples (sample size 33 mm × 40 mm) were positioned to rotate by 360° during scanning with a pixel (sic) size of 22.40 µm, resulting in a total scanning time of 65 min per sample. Initial 2D radiographs (averaged from 2 scans) were obtained at every 0.13° rotation. PerGeos software (Thermo Fisher Scientific) was used for pre-processing of the XMT data (3D visualization, segmentation) and porosity analysis, whereas the open source image processing software ImageJ [28] with the extension BoneJ [29] was used for analysis of trabecular geometries, i.e., thickness of pore walls in the samples. Four different solid parts from each sample were chosen from a stack of XMT images for 3D pore morphology analysis (Figure 1). The connectivity index was determined by dividing the volume of the largest pore by the total pore volume. This index can be considered as the volumetric fraction of open cells [30]. Samples were scanned and analysed in triplicate.

### 2.9. Sensory Analysis

Sensory analysis was performed for single-layer printed and baked samples (R, R1M1, and M). Sensory evaluations were carried out by a 10-member trained panel with proven skills at the sensory laboratory of Technical Research Centre of Finland Ltd. (VTT), which fulfils the requirements of the ISO standards [31,32]. The sensory method was descriptive analysis [33], and attributes related to the appearance, texture and taste/flavour were evaluated. The attribute intensities (0–10) were rated on continuous graphical intensity scales, verbally anchored from both ends, where 0 = attribute not existing and 10 = attribute very clear. The sample snacks were coded with three-digit numbers, and served to the assessors in random order in two replicate sessions from odourless disposable containers covered by a lid. The panellists were instructed not to swallow the samples. Water was provided to the assessors for cleansing the palate between the samples. The scores were recorded and collected using a computerized Compusense Five data system, Ver. 5.6 (Compusense, Guelph, ON, Canada).

The protocol for performing the sensory evaluation was accepted by the Ethical Committee of VTT. Panellists were VTT employees belonging to in-house food and beverage sensory panel. In accordance with the European Union (EU) General Data Protection Regulation GDPR (2016/679), necessary individual information of the panel members was collected in the data protection registry, and the panellists have given their consent for this.

### 2.10. Statistical Analysis

Statistical differences between samples was assessed by one-way analysis of variance (ANOVA) and samples grouped based on post hoc testing with Dunnett’s T3 for samples with unequal variances and Tukey’s honestly significant difference (HSD) for samples with equal variance using a significance level of 0.05. Sensory data were subjected to multivariate analysis of variance where individual means were identified by Tukey’s test (*p* < 0.05). All statistical analysis were made by using IBM SPSS Statistics, Ver. 25 (IBM Corporation, New York, NY, USA).

## 3. Results

### 3.1. Rheological Characterisation of Printing Pastes

**Figure 2:**
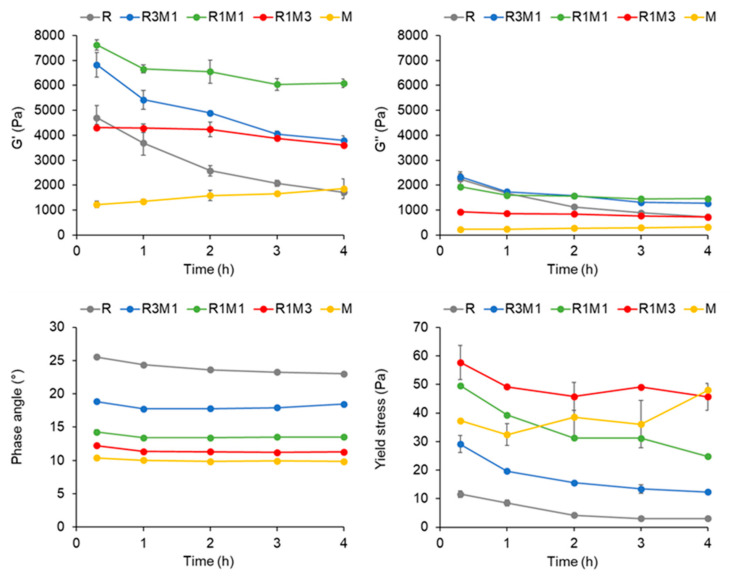
Changes in viscoelastic properties, i.e., G’, G’’ and phase angle in linear viscoelastic region and yield stress, of printing pastes over a 4 h period after preparing the pastes. R, the rye flour-based paste; M, milk powder-based paste; R1M3, paste composed of 25% rye flour and 75% milk powder; R1M1, paste composed of 50% rye flour and 50% milk powder; R3M1, paste composed of 75% rye flour and 25% milk powder.

The viscoelastic properties of the pastes used in 3D printing were examined by oscillatory stress sweep measurements. All printing pastes showed elasticity-dominating viscoelastic behaviour (G’ > G’’ and phase angle < 45°) shortly after paste preparation (Figure 2). The storage modulus (G’), a measure of the mechanical rigidity of the material at rest, of all pastes was in the range of 1000–8000 Pa. The milk powder-based paste (M) had the lowest G’ and inclusion of rye flour up to 50% increased the G’, whereas higher amounts of rye flour caused a decrease in G’. A high G’ seemed to be linked with a high carbohydrate content (Table 1) in the paste. The phase angle was the lowest, indicating the highest degree of elasticity, for the milk powder-based paste (M) shortly after preparation. The gradual increase of rye flour supplementation levels from 0 to 100%, accompanied by increased moisture content from 42% to 58%, increased the phase angle values from 10° to 26°. The yield stress, indicating the point at which the network structure of a material starts to break down and flow is initiated, varied between 10 and 60 Pa, with the rye flour-based paste (R) having the lowest yield stress and the paste composed of 25% rye flour and 75% milk powder (R1M3) the highest.

Although the viscoelastic properties of the pastes slightly varied during 4 h storage at room temperature (Figure 2), they were still printable. Samples with high rye flour content (R and R3M1) showed the most significant changes in the determined rheological properties over time, probably due to the activity of endogenous enzymes (xylanase and α-amylase) in the wholegrain rye flour causing solubilisation of the cell wall material and degradation of starch [34] with a concomitant decrease in G’, G’’ and yield stress. The milk powder-based paste (M) showed fewer changes over time, only a small increase in G’, G’’ and yield stress was observed, likely due to increased hydration of the milk powder over time.

### 3.2. Characterisation of Printed Single-Layer Samples

#### 3.2.1. Appearance and Weight

**Figure 3:**
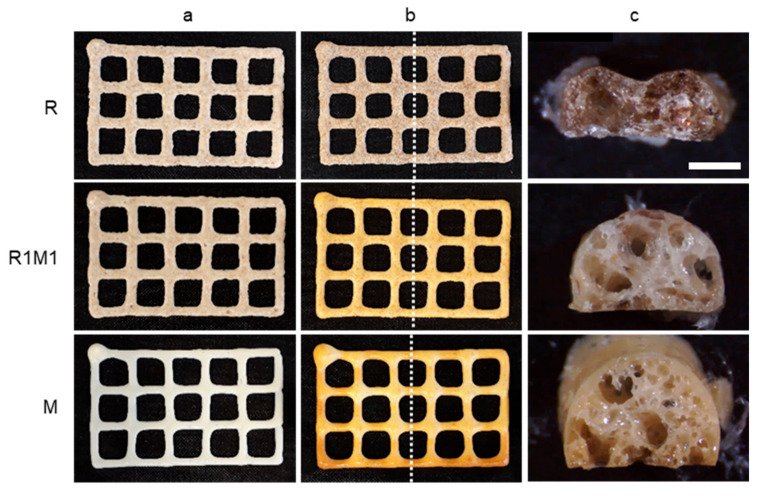
Appearance of 1-layer samples after (**a**) printing and **(b**) baking. The cutting position in the hardness measurement is marked in the baked samples with a vertical dotted line. The cross-sectional images (**c**) were taken from the cut surface (scale bar 1 mm).

Pictures of printed single-layer samples are presented in Figure 3 before (a) and after baking (b), showing that all samples retained the printed grid-like pattern well during baking. The milk powder-containing samples showed the most pronounced change in colour during baking. The cross-sectional images in Figure 3c revealed that the presence of milk powder caused substantial expansion of the printed samples during baking. The sample consisting of purely rye flour (R) had a more flat appearance, with the two adjacent strands of printed material clearly visible in the cross-sectional image.

The reproducibility of the printing and baking process was followed by weighing the samples right after each process. The standard deviation in weight of replicate samples of one material was at maximum 11% of the mean value (Table 2), showing a decent level of reproducibility of the printing and baking process. There was a slight difference in the average weight of the samples printed from different pastes, but that was intentional in order to obtain samples of equal weight and dry matter content after a constant baking time (from pastes with varying water content). This approach was successful as the variation in weight of the baked and stored samples from the different materials was rather small (Table 2).

#### 3.2.2. Microstructure

**Figure 4:**
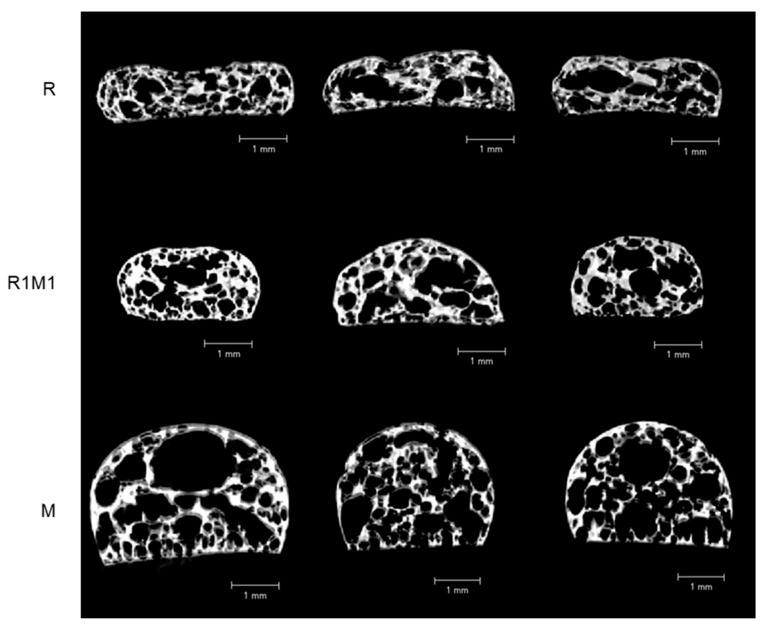
Cross-sectional 2D X-ray micro tomography images of printed single-layer samples after baking. The images represent the area of the sample where the hardness measurement by cutting was performed.

**Figure 5:**
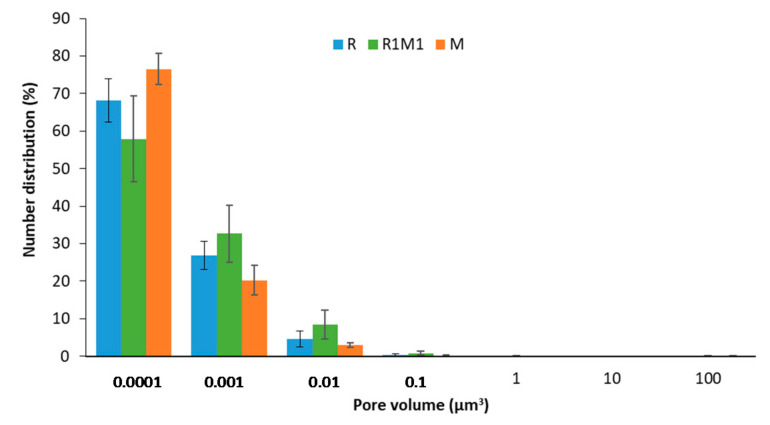
Pore size distribution in single-layer printed samples after baking.

The microstructure of the printed samples after baking was investigated with stereomicroscopy and XMT. Milk powder-containing samples had a more expanded structure than the rye flour-based samples based on both stereomicroscopy (Figure 3c) and 2D XMT (Figure 4) images. The overall porosity calculated from XMT images was the highest for M (59%) samples followed by R1M1 (49%) and R (45%) samples (Table 3). Cell wall thickness was also analysed as it may affect the fracture behaviour of samples during hardness measurements [35], but no significant difference in cell wall thickness was observed between samples (Table 3). The high values of the connectivity index (Table 3) suggest that all samples had an open pore structure. The pore size distribution was similar for all samples (Figure 5).

#### 3.2.3. Texture Analysis

The fracture force, describing the cutting force needed to break the samples, of the baked single-layer samples are presented in Table 4. The fracture force of the samples containing both milk powder and rye flour (R1M1) was significantly higher than the fracture force of the samples containing only rye flour (R) or milk powder (M).

#### 3.2.4. Sensory Analysis

**Figure 6:**
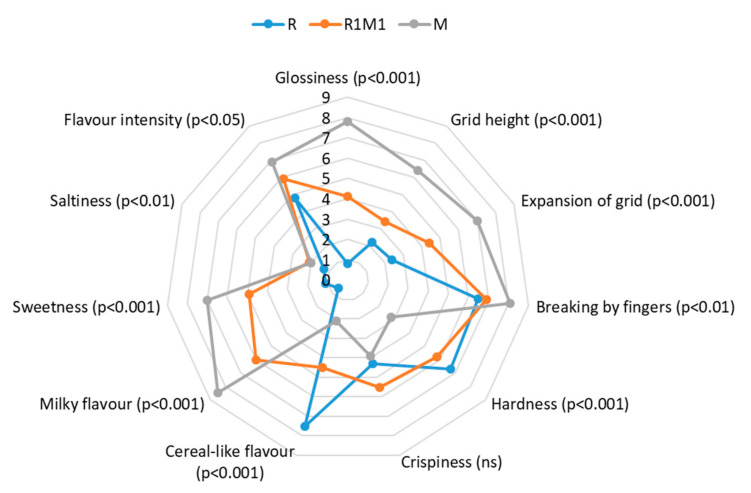
Results of the sensory profiling (*n* = 2 × 10) of the printed and baked single-layer samples. The level of the statistical significances of the difference between samples is marked by the *p*-value or ns (not significant).

The appearance, texture and flavour of the baked single-layer samples were evaluated by sensory analysis. There were statistically significant differences between the samples in all evaluated attributes except for crispiness (Figure 6). The whole milk sample (M) was most glossy (*p* < 0.001), had most expanded grids (*p* < 0.001), was easiest to break (*p* < 0.01), and had the most intense milky flavour (*p* < 0.001) and highest sweetness (*p* < 0.001). The rye flour sample (R) was least glossy, had lowest grid height (were flat), was hardest to break together with whole milk-rye flour sample (R1M1), was most cereal-like (*p* < 0.001), least sweet and salty. The R1M1 sample, which consisted of equal amounts of rye flour and milk powder, was located between these two samples in terms of sensory characteristics.

### 3.3. Characterisation of Printed 5-Layer Samples

#### 3.3.1. Appearance and Weight

**Figure 7:**
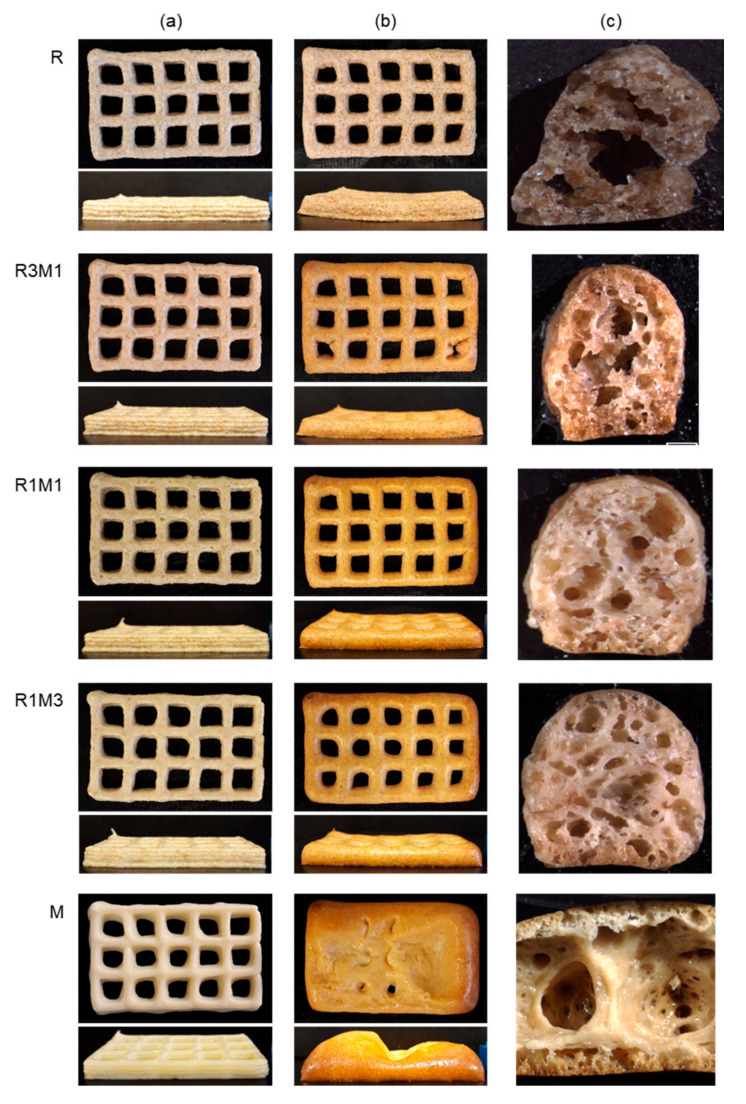
Appearance of 5-layer samples after printing (**a**) and baking (**b**). Stereomicroscopic images of sample cross-sections are presented in panel (**c**).

All pastes performed well in multi-layered printing, in terms of shape accuracy and stability after printing (Figure 7a). However, post-processing of 5-layered samples by baking (Figure 7b) was not as successful as with the single-layer samples. The worst result was obtained with the sample containing solely milk powder (M), which expanded a lot during baking, causing a total collapse of the printed shape after baking. Increasing the amount of rye flour in the printing paste prevented excessive expansion and resulted in better shape stability. A clearly improved shape stability after baking was already obtained by replacing 25% of the milk powder with rye flour (sample R1M3), but even better shape stability was found for the 50–50 mixture (R1M1). Samples made with only rye flour (R) tended to shrink during baking, resulting in bending of the sample. According to stereomicroscopy images (Figure 7c), the most homogeneous pore structure was obtained for R1M3 and the pore size was found to gradually increase with an increase in rye flour content. An exception was the completely collapsed sample M that was occupied mainly with large pores covered with a thick crust consisting of small pores.

The reproducibility of the printing process was on a rather good level for the 5-layer samples, as the standard deviation in sample weight after printing (Table 5) was 6% of the mean value at maximum. The inclusion of milk powder to the rye paste improved reproducibility in multi-layer printing. The aim was to obtain samples of equal weight and dry matter content (and dimensions) after baking and storage, but that was not fully reached as there were some variations in the weight (5.4–6.5 g, Table 5) and dry matter content (92.1–93.5%, Table 6) of the final samples after baking and storage.

#### 3.3.2. Texture Analysis

The force required to fracture the printed samples after baking was a lot higher for the 5-layer (Table 6) than for the single-layer (Table 4) samples. The 5-layer samples containing 100% or 75% rye flour (R and R3M1) had a clearly lower fracture force than those containing 50% or less rye flour (R1M1 and R1M3). For the 5-layer samples, the highest fracture force was measured for the sample containing equal amounts of rye flour and milk powder (R1M1), a similar finding as for the single-layer samples. The fracture force was not measured for the totally collapsed 100% milk powder sample (M) as its shape differed so much from the other samples.

## 4. Discussion

In this study, we have shown that whole milk powder and wholegrain rye flour and their mixtures are suitable materials for extrusion-based 3D printing. The benefits of whole milk powder for 3D food printing are its small particle size, good dispersibility in water and presence of fat. Wholegrain rye flour, on the other hand, is a very good source of dietary fibre in 3D printing formulations due its low gluten content [36]. Gluten is responsible for the generation of the extensible and elastic character of wheat-based doughs, both of which are unwanted properties in paste extrusion-type 3D printing. Low-gluten wheat flour has been used in some 3D printing studies [14,24] to assure proper printability.

Rheological measurements have proved useful in predicting the suitability of food formulations for extrusion-based 3D printing. In several studies a link between viscosity, storage modulus G’ or yield point (yield stress) of printing pastes and the printability (extrusion pressure, shape stability) has been suggested [4,7,21,37]. The relationship between rheological properties and printability was studied in a more systematic manner by Zhu et al. [20]. They showed that the flow stress (yield point), defined as the crossover of G’ and G’’ in a stress sweep measurement, correlated positively both with extrusion force and the estimated stress at collapse (proportional to object height) for commercial food materials such as tomato puree, mayonnaise, meat and vegetable-based spreads. However, for pastes containing a high amount fat and solid particles the relation was less obvious. In another study, it was concluded that the storage modulus (G’) together with the damping factor (G’’/G’) of a material were more important than the yield stress in defining the dimensional stability of a cylindrical structure made of hydroxypropyl methylcellulose, wheat starch or gelatine [38].

In our study, all tested formulations showed good printability and dimensional stability after printing. We determined the rheological properties of the formulations by stress sweeps measurements in a similar manner as in the study of Zhu et al. [20]. The G’ values (1000–8000 Pa) measured for the printing pastes in our study were at the same level as those measured by Zhu et al. for tomato pastes from which 3D printing of at least 60 mm high hollow square columns (square side 30 mm) was possible with a 1.2 mm nozzle. On the other hand, Nijdam et al. [38] showed that a G’ value of higher than 10,000 Pa was not enough to guarantee dimensional stability of a 2 cm high gel cylinder (diameter 2.9 cm) made of 10% hydroxypropyl methylcellulose. The phase angle (δ) values presented in Figure 2 can be compared with the damping factor (G’’/G’) values presented by Nijdam et al. [38] as the phase angle also represents the dependence of G’’ with G’ (tan δ = G’’/G’). The G’’/G’ values in our study varied between 0.17 and 0.48. The lowest values were obtained for the milk powder-based paste (M) and the highest for the rye flour-based paste (R), which according to Nijdam et al. [38] could indicate that the milk powder-based paste showed better dimensional stability in printing than the rye-flour based paste. However, in our previous study [4], we showed that two printing pastes having similar G’ and phase angle values were different in terms of shape stability after printing. The more shape-stable material had a clearly higher yield stress than the less stable one, which suggests that the yield stress could be of importance in defining the deformability of printed structures.

Zhu et al. [20] presented a linear correlation between the flow point and the collapsing height of 3D printed tomato paste structures. The flow point values were defined as the crossover point of G’ and G’’ in the stress sweep measurement. The flow point can be considered as a yield point of a material in a similar way as the yield stress determined in our study, although our definition was somewhat different. The yield stress values (10–60 Pa) in our study were lower than the flow stress values (>120 Pa) presented by Zhu et al. [20]. It could still be expected that the pastes with a higher yield stress in our study, i.e., those containing at least 50% milk powder, could show a higher rigidity after printing than those with less milk powder.

An interesting finding in our study was that G’ did not show a clear relationship with the content of rye flour or milk powder in the printing paste, but seemed to increase with an increasing carbohydrate content of the printing paste. The other rheological parameters of interest, G’’, phase angle and yield stress, were more clearly linked to the contents of rye flour or milk powder in the formulation. A change in these parameters towards a direction considered more beneficial for shape stability during printing, i.e., decrease in G’’ and phase angle and increase in yield stress, could be realised by increasing the milk powder and decreasing the water content of the formulation.

The formulations used in extrusion-based 3D printing are typically concentrated multiphase dispersions showing complex rheological behaviour. Rheological characterization of such materials is often complicated as their properties vary by time and shear-history. Artefacts are also possible in rheometer measurements due to the presence of large particles, wall depletion, slip or non-homogeneous flow fields [39,40,41]. Thus, the prediction of the printability of a material by rheological measurements might be less straightforward than anticipated. Also other factors, such as homogeneity, particle size and air content of printing paste contribute to the quality of the printed filament [23].

Although all formulations in our study showed good shape stability after printing, not all of them retained their shape after post-processing by baking. The 5-layer samples printed from milk powder-based paste (M) collapsed totally due to extensive expansion of the material during baking at 150 °C. The single-layer samples from milk powder also expanded during baking, but the expansion was more controlled due to faster drying and hardening of the thin sample. The rye flour samples kept their shape better during baking, but showed a slight tendency to shrink. The different behaviour of these materials during baking can be related to the difference in their composition. Milk powder contains a high amount of fat, which is partly crystalline at room temperature [42]. The majority of the fat crystals melt upon heating to 40 °C, which decreases the viscosity of the system and can already at that temperature cause deformation of the printed shape. The viscosity of the rye flour paste is at first expected to slightly decrease with increasing temperature (due to decreasing viscosity of water) but to increase greatly when the gelatinization temperature of starch is reached, which could be a reason for the better dimensional baking stability of the rye flour-based samples. Measuring the rheological properties of the materials during heating could have given more insight into the reasons for their different baking stability, although the baking process cannot fully be simulated in a rheometer. Kim et al. [27] for example showed that the improved baking stability of cookie dough with xanthan gum addition was related to a higher rigidity (G’) of the dough during heating. In addition to heat-induced changes in rheological properties, also the process of water evaporation has to be considered when printed samples are heated to above 100 °C. The expansion of the milk-based samples was most likely caused by water evaporation. It is possible that the surface-active proteins in milk stabilised the gas bubbles formed by water evaporation and thereby enhanced bubble growth. Rye flour also contains surface-active proteins [43], but it is possible that the fibre particles present in the material hamper bubble growth in a similar way as in bread baking [44]. This theory is supported by the higher porosity of the milk-based samples as determined with X-ray tomography (Table 3) and might be one reason why the addition of rye flour improved the baking stability of the milk-based system. A benefit of 3D printing is its ability to create low fill density and large surface area products in which water evaporation during baking is more effective than in more dense conventional products [45].

Instrumental texture measurements of baked 3D printed objects are challenging due to difficulties in producing samples with similar dimensions and dry matter content, which is a prerequisite for obtaining reproducible results. Our measurements, however, gave an indication that single-layer samples made from a mixture of milk and rye flour (R1M1) had a markedly higher fracture force (hardness) than those made from solely milk or rye flour. The high force required to fracture sample R1M1 cannot solely be explained by a difference in composition, porosity or grid height/volume as those were on an intermediate level for sample R1M1 as compared with samples R and M. The fracturability of the samples could also have been affected by their dry matter content or pore cell wall thickness, but sample R1M1 did not significantly differ from the other samples in these properties either. For the 5-layer samples, the highest fracture force was also measured for the R1M1 sample. The results of the 5-layer samples, however, do not help much in evaluating the reasons for the high fracture force of sample R1M1, as the texture of sample M could not be measured due to its complete collapse during baking. It is likely that the texture of the baked samples was affected by a combination of several of the properties mentioned, e.g., the composition of the solid phase together with an intermediate porosity. It is also possible that rye fibre acted as a reinforcing agent in the milk-rye composite [46] and that this effect was at an optimal level in the 50–50 mixture.

The texture-related attributes evaluated for the single-layer samples by sensory evaluation were not fully in line with the instrumental measurements. According to sensory analysis, the milk powder- based sample (M) was most difficult to break by bending by fingers. Sample (R1M1), which required the highest fracture in the instrumental measurement, was perceived to be the hardest in the mouth, but sample R was perceived to be as hard as sample R1M1. In the sensory evaluation, the force required to break the sample (by fingers) and the hardness as perceived in the mouth were affected in opposite ways by an increased level of rye flour in the formulation; breaking force was decreased and hardness in the mouth increased. The hardness in the mouth of the milk powder-containing samples was most likely affected by melting of milk fat at body temperature. The discrepancy between the instrumental and sensory texture results in our study once again demonstrates the difficulty of obtaining full textural characterisation of a food product by a single method.

Sensory analysis gave valuable information also about the appearance and flavour of the baked single-layer samples. The presence of milk powder increased the grid height and the expansion of the grids, which was in line with the porosity results obtained by X-ray micro tomography. Milk powder also increased the glossiness of the samples, most likely due to the fat present in the material. The sweetness and flavour intensity of the samples increased with a higher amount of milk in the formulation. Inclusion of milk powder to a rye-base could also in this respect make the products more appealing to the consumer.

Using a mixture of milk powder and wholegrain rye flour in a snack formulation could be beneficial also from a nutritional point of view. The approximate composition of the baked samples produced in our study is shown in Table 7. For example with a 50–50 mixture of milk and rye (R1M1), a snack product with a relatively high protein and dietary fibre content could be formed. The fat content is rather high, but it could be lowered by using a milk powder with a somewhat lower fat content.

## 5. Conclusions

This study addressed the potential of 3D printing as a processing technology for delivering personalized healthy eating solutions to consumers. A mixture of milk powder and wholegrain rye flour showed great potential as a formulation for protein- and dietary fibre-rich snack products produced by extrusion-based 3D printing in combination with baking. Trials with various ratios of milk powder and rye flour in the printing formulation revealed that although all formulations showed good printability and shape stability after printing, the shape retention during baking at 150 °C was a challenge with samples containing a high amount of milk powder. The extensive expansion of the milk powder-based samples during baking could be reduced by the inclusion of rye flour to the formulation. Milk powder gave volume and glossiness to the baked samples, whereas rye flour seemed to increase the baking stability and rigidity. The concept of delivering customised foods to consumers by 3D printing is, however, still in an early phase of development and more work is needed to tackle technological difficulties related to the 3D printing of food materials.

## Figures and Tables

**Table 1:** Approximate composition of printing pastes (calculated based on raw material composition given by manufacturer).

Paste Code	Rye to Milk Ratio	Paste Composition (%)				
		Protein	Carbohydrates	Dietary Fibre	Fat	Water
R	1:0	4.7	26.8	9.4	1.1	58
R3M1	3:1	7.4	27.7	7.9	4.1	53
R1M1	1:1	10.5	28.1	5.8	7.7	48
R1M3	1:3	13.5	27.0	3.1	11.4	45
M	0:1	16.8	25.6	0	15.6	42

**Table 2:** Weight of single-layer samples right after printing, baking and storage (*n* ≥ 17).

Sample	After Printing (g)	After Baking (g)	After Storage (g)
R	2.2 ± 0.1 ^a^	1.3 ± 0.1 ^a^	1.1 ± 0.1 ^a^
R1M1	2.0 ± 0.2 ^b^	1.2 ± 0.1 ^a^	1.1 ± 0.1 ^b^
M	1.9 ± 0.2 ^b^	1.2 ± 0.1 ^a^	1.2 ± 0.1 ^b^

Different letters within the same column indicate significant difference (*p* < 0.05). R, the rye flour-based paste; M, milk powder-based paste; R1M1, paste composed of 50% rye flour and 50% milk powder.

**Table 3:** Microstructural properties of single-layer printed samples after baking based on analysis of 3D X-ray micro tomography images.

Sample	Porosity (%)	Cell Wall Thickness		Connectivity Index (%)
		Mean (mm)	Maximum (mm)	
R	45 ± 4 ^a^	0.14 ± 0.01 ^a^	0.33 ± 0.03 ^a^	97 ± 2 ^a^
R1M1	49 ± 3 ^b^	0.14 ± 0.01 ^a^	0.35 ± 0.04 ^a^	96 ± 2 ^a^
M	59 ± 3 ^c^	0.13 ± 0.02 ^a^	0.36 ± 0.08 ^a^	99 ± 0 ^b^

Different letters within the same column indicate significant difference (*p* < 0.05).

**Table 4:** Fracture force and dry matter content of printed single-layer samples after baking and storage.

Sample	Fracture Force (*n*)	Dry Matter Content (%)
R	12 ± 2 ^a^	91.6 ± 1.0 ^a^
R1M1	20 ± 5 ^b^	93.5 ± 0.4 ^b^
M	11 ± 1 ^a^	93.0 ± 0.7 ^b^

Different letters within the same column indicate significant difference (*p* < 0.05).

**Table 5:** Weights of 5-layer samples after printing, baking and storage (*n* = 6). Different letters within the same column indicate significant difference (*p* < 0.05).

Paste	After Printing (g)	After Baking (g)	After Storage (g)
R	11.4 ± 0.7 ^a^	7.4 ± 0.6 ^a^	5.4 ± 0.3 ^a^
R3M1	11.5 ± 0.6 ^a^	7.7 ± 0.5 ^ab^	6.0 ± 0.3 ^ab^
R1M1	11.2 ± 0.4^a^	8.3 ± 0.4 ^ab^	6.3 ± 0.2 ^bc^
R1M3	10.7 ± 0.3 ^ab^	8.3 ± 0.3 ^b^	6.5 ± 0.2 ^c^
M	10.1 ± 0.3 ^b^	8.3 ± 0.3 ^b^	6.4 ± 0.1 ^c^

**Table 6:** Fracture force and dry matter content of printed 5-layer samples after baking and storage.

Sample	Fracture force (*n*)	Dry Matter Content (%)
R	83 ± 16 ^a^	93.4 ± 0.5 ^ab^
R3M1	85 ± 27 ^a^	93.5 ± 0.7 ^b^
R1M1	348 ± 14 ^b^	92.7 ± 0.4^ac^
R1M3	305 ± 21 ^c^	92.5 ± 0.6 ^c^
M	-	92.1 ± 0.3 ^c^

Different letters within the same column indicate significant difference (*p* < 0.05).

**Table 7:** Approximate composition (%) of 3D printed 5-layer samples after baking (calculated based on average dry matter content and raw material composition given by manufacturer).

Sample	Protein (%)	Carbohydrates (%)	Dietary Fibre (%)	Fat (%)	Moisture (%)
R	10.5	59.6	20.9	2.4	6.6
R3M1	14.6	55.1	15.7	8.1	6.5
R1M1	18.6	50.0	10.4	13.7	7.3
R1M3	22.7	45.3	5.2	19.2	7.5
M	26.7	40.6	0.0	24.8	7.9
